# Sequencing of Seven Haloarchaeal Genomes Reveals Patterns of Genomic Flux

**DOI:** 10.1371/journal.pone.0041389

**Published:** 2012-07-24

**Authors:** Erin A. Lynch, Morgan G. I. Langille, Aaron Darling, Elizabeth G. Wilbanks, Caitlin Haltiner, Katie S. Y. Shao, Michael O. Starr, Clotilde Teiling, Timothy T. Harkins, Robert A. Edwards, Jonathan A. Eisen, Marc T. Facciotti

**Affiliations:** 1 Microbiology Graduate Group, University of California Davis, Davis, California, United States of America; 2 Faculty of Computer Science, Dalhousie University, Halifax, Nova Scotia, Canada; 3 Genome Center, University of California Davis, Davis, California, United States of America; 4 Children’s Hospital Oakland Research Institute, Oakland, California, United States of America; 5 Department of Forensic Science, University of California Davis, Davis, California, United States of America; 6 Davis Senior High School, Davis, California, United States of America; 7 454 Life Sciences, a Roche Company, Branford, Connecticut, United States of America; 8 Life Technologies, Beverly, Massachusetts, United States of America; 9 Department of Computer Science, San Diego State University, San Diego, California, United States of America; 10 Department of Biology, San Diego State University, San Diego, California, United States of America; 11 Division of Mathematics and Computer Science, Argonne National Laboratory, Argonne, Illinois, United States of America; 12 Department of Evolution and Ecology, University of California Davis, Davis, California, United States of America; 13 Department of Medical Microbiology and Immunology, University of California Davis, Davis, California, United States of America; 14 Department of Biomedical Engineering, University of California Davis, Davis, California, United States of America; Max-Planck-Institute for Terrestrial Microbiology, Germany

## Abstract

We report the sequencing of seven genomes from two haloarchaeal genera, *Haloferax* and *Haloarcula*. Ease of cultivation and the existence of well-developed genetic and biochemical tools for several diverse haloarchaeal species make haloarchaea a model group for the study of archaeal biology. The unique physiological properties of these organisms also make them good candidates for novel enzyme discovery for biotechnological applications. Seven genomes were sequenced to ∼20×coverage and assembled to an average of 50 contigs (range 5 scaffolds - 168 contigs). Comparisons of protein-coding gene compliments revealed large-scale differences in COG functional group enrichment between these genera. Analysis of genes encoding machinery for DNA metabolism reveals genera-specific expansions of the general transcription factor TATA binding protein as well as a history of extensive duplication and horizontal transfer of the proliferating cell nuclear antigen. Insights gained from this study emphasize the importance of haloarchaea for investigation of archaeal biology.

## Introduction

In recent years, the Archaea have been shown to play major roles in global element cycling [1]–[6], form animal-archaeal symbiosis with potential medical importance [7], possess metabolic pathways unknown to the other two domains of life [3], [8], [9] and produce stress-resistant enzymes with great potential for a variety of industrial applications [10], [11]. These discoveries emphasize the importance of expanding our knowledge of this third domain of life.

The family *Halobacteriacea* (also known as the Haloarchaea) in the phylum *Euryarchaeota* has enormous potential to serve as a model group for the study of archaeal biology. As aerophilic mesophiles, many haloarchaea are easily cultivated in the laboratory, making them one of the most widely studied archaeal groups and leading to the development of a variety of biochemical, genetic and genomic tools for several diverse haloarchaeal species. Although united by their ability to thrive at high salinities, haloarchaea possess a wide range of physiologies (including alkaliphiles, facultative thermophiles, thermoalkaliphiles, and psychrotolerant species) [12] and diverse metabolic strategies [13], making them model organisms for exploring archaeal biology.

In addition to serving as a model group for study of the Archaea in general, the Haloarchaea possess unique properties making them important objects of study in their own right. For example, understanding the genetic basis for these organisms’ ability to thrive in hypersaline environments (∼3–5 M salts) will inform efforts to develop salt-tolerant crop plants for growth in currently non-arable land. The Haloarchaea are also promising sources of salt and ionic liquid tolerant enzymes for various industrial processes, including biofuels manufacturing [14]–[17], [11] and bioplastics production.

As of September 2011, the National Center for Biotechnology Information (NCBI) lists 1,628 completed bacterial, 37 eukaryotic and 116 archaeal genomes [http://www.ncbi.nlm.nih.gov/genomes]. This discrepancy in the number of available bacterial and archaeal genomes is particularly striking when one considers that archaeal and bacterial genomes are similar in size and coding density, both lacking the extensive DNA repeats which complicate sequencing and assembly of eukaryotic genomes. Of the 116 completely sequenced archaeal genomes, fourteen are from the Haloarchaea, spanning thirteen genera, making this one of the most deeply sequenced archaeal clades. These haloarchaeal genomes average 3.3 Mbp, ranging from 2.67–5.44 Mbp. Distinguishing features of haloarchaeal genomes include multiple replicons, high GC content (averaging >60%), and highly acidic proteomes (pI ∼5), thought to be an adaptation to life at low water activity [18].

While published and in-progress haloarchaeal genomes have yielded insights into the biology and evolution of these organisms [19]–[21], these sequenced genomes represent only a small sampling of this large and phenotypically diverse group. Recent studies have shown that deep sequencing within a phylogenetic group can yield insights into mechanisms of evolutionary and functional diversification [22]. We report here the sequencing of seven novel haloarchaeal genomes. To allow for both depth and breadth of sequencing and to facilitate comparative analysis, we elected to sequence multiple species from each of two haloarchaeal genera (*Haloferax* and *Haloarcula*), each with a previously sequenced member (Table 1). Here we present our analysis of these genomes, with comparison to previously sequenced haloarchaea.

**Table 1:** Genomes included in this study.

Organism	Ascension #s	Reference
*Haloferax mucosum* ATCC BAA-1512	PubSEED: 662479.5	This study
*Haloferax denitrificans* ATCC 35960	PubSEED: 662478.4	This study
*Haloferax sulfurifontis* ATCC BAA-897	PubSEED: 662480.4	This study
*Haloferax mediterranei* ATCC 33500	PubSEED: 523841.6	This study
*Haloferax volcanii* DS2	GenBank: CP001953.1–CP001957.1	[19]
*Haloarcula californiae* ATCC 33799	PubSEED: 662475.4	This study
*Haloarcula marismortui* ATCC 43049	GenBank: AY596290.1–AY596298.1	[18]
*Haloarcula sinaiiensis* ATCC 33800	PubSEED: 662476.5	This study
*Haloarcula vallismortis* ATCC 29715	PubSEED: 662477.4	This study
*Halorubrum lacusprofundi* ATCC 49239	GenBank: CP001365.1–CP001367.1	None
*Halomicrobium mukohataei* DSM 12286	GenBank: CP001688.1, CP001689.1	[23]
*Halorhabdus utahensis* DSM 12940	GenBank: CP001687.1	[24]
*Natronomonas pharaonis* DSM 2160	GenBank: CR936257.1–CR936259.1	[25]
*Halobacterium salinarum* R1	GenBank: AM774415.1-AM774419.1	[26]
*Halobacterium sp* NRC-1	GenBank: AE004437.1, AF016485.1, AE004438.1	[27]
*Haloquadratum walsbyi* DSM 16790	GenBank: AM180088.1, AM180089.1	[28]
*Halogeometricum borinquense* DSM 11551	GenBank: CP01690.1–CP01695.1	[29]
*Halalkalicoccus jeotgali* B3	GenBank: CP002062.1–CP002068.1	[30]
*Natrialba magadii* ATCC 43099	GenBank: CP001932.1–CP001935.1	[31]
*Haloterrigena turkmenica* DSM 5511	GenBank: CP001860.1–CP001866.1	[32]
*Halopiger xanaduensis* SH-6	GenBank: CP002839.1–CP002842.1	[33]

## Results and Discussion

### Genome Features and Cross-genera Comparisons

#### A single plate of sequencing provides deep coverage of eight archaeal genomes

**Figure 1:**
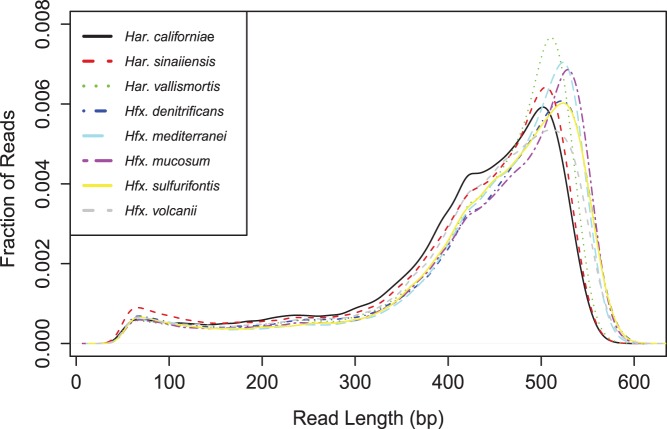
Read length. Distribution of read-length in seven newlysequenced haloarchaeal genomes and one sequencing control (*Haloferax volcanii*). After quality-trimming, mean read-length for each genome was between 410 and 439 base pairs.

Eight species of the family Halobacteriacea - three Haloarcula (Har. californiae, Har. sinaiiensis, Har. vallismortis) and five Haloferax (Hfx. denitrificans, Hfx. mediterranei, Hfx. mucosum, Hfx. sulfurifontis, and Hfx. volcanii) - were sequenced on a single GS FLX Titanium run, with the previously sequenced Haloferax volcanii included as a control. After removal of low-quality nucleotides, mean read-length for each genome was between 410 and 439 base pairs (Figure 1). Sequencing depth ranged from 19 to 29x, and assembly resulted in 21–168 contigs over 200 bp in length per genome with Hfx. species assembling on average 1.8×better than Har. species (Table 2).

**Table 2:** Genome characteristics.

Organism	#Contigs >200 bp	Assembled bp	Coverage	CDSs	% w/Function	RNAs	% Coding	%GC	Isolated From
*Haloarcula californiae*	168	4,420,514	21	4627	62.5	69	87.00	60.82	Baja, Mexico
*Haloarcula sinaiiensis*	140/10*	4,524,388	19/45*	4538	63.1	55	84.62	60.77	Red Sea, Israel
*Haloarcula vallismortis*	88	3,930,055	24	4084	65.2	84	88.22	61.79	Death Valley, California, USA
*Haloarcula marismortui* (GenBank)	9	4,274,642	N/A	4325	51.8	62	85.83	61.12	Dead Sea
*Haloferax denitrificans*	21	3,848,468	25	3809	70.5	58	85.80	66.27	San Francisco Bay, California, USA
*Haloferax mediterranei*	141/5*	3,905,749	26/53*	3942	65.7	62	85.83	60.27	Alicante, Spain
*Haloferax mucosum*	26	3,371,699	29	3455	66.0	61	86.38	61.84	Shark Bay, Australia
*Haloferax sulfurifontis*	29	3,816,558	27	3856	67.6	59	86.56	66.30	Zodletone spring, SW Oklahoma, USA
*Haloferax volcanii* (resequenced)	145	3,920,004	25	N/A	N/A	N/A	N/A	N/A	Shore mud, Dead Sea
*Haloferax volcanii* (GenBank)	5	4,012,900	N/A	4015	63.3	49	85.56	65.48	Shore mud, Dead Sea

Note: *Indicates results of mate-pair sequencing.

Note: The *Haloferax volcanii* control was not submitted to IMG for annotation and so some features are N/A.

There were thirteen single base call discrepancies between the *Haloferax volcanii* sequencing control and the published genome for this species, for an average of one differently called base per 305 Kbp. In addition, the newly assembled *Hfx. volcanii* genome was missing 123 regions present in the published genome, with a median length of 1126 bp and a total length of 119,245 bp of missing sequence. The sequencing control also had 140 short sequences not present in the published genome with a median length of 3 bp and a total of 1,319 bp additional sequence. While it is tempting to use this data to calculate error rates for the sequencing technology used in this study, we can not exclude the possibility that a portion of the differences between these two assemblies may be due to natural variation within the *Haloferax volcanii* DS2 population, errors in the previously published genome, and/or evolution of this organism between sequencing efforts.

To determine the benefit of paired-end sequencing to improving assembly, additional 8 Kb span paired-end libraries were sequenced for one species of each genus, *Har. sinaiiensis* and *Hfx. mediterranei*, resulting in assembly of *Hfx. mediterranei* into five scaffolds. As the previously sequenced member of this genus, *Hfx. volcanii*, possesses four chromosomes and a plasmid [19], we suspected that the assembly of *Hfx*. *mediterranei* into five scaffolds may indicate complete assembly for this species. To test whether our scaffolds represented complete replicons, we designed primers to the ends of each scaffold and looked for formation of short PCR products, indicating circularization of the replicon near our scaffold ends (for primer list see Table S1). Scaffolds 4 (322 Kbp), and 5 (132 Kbp) were shown to be complete, and scaffold 3 (504 Kbp) was found to be missing approximately one Kbp of sequence. Our PCR-based test could not conclusively determine whether the two remaining scaffolds (scaffold 2 (2.9 Mbp) and scaffold 1 (2.2 Kbp)) were complete (Figure S1).

The assembly of *Har. sinaiiensis* was similarly improved from 140 contigs to 10 scaffolds (Table 2). While it is possible that this may represent full assembly, as a previously sequenced member of this genus, *Har. marismortui*, possesses nine replicons [18], several of the *Har. sinaiiensis* scaffolds are very small (below 5 Kbp) while the smallest plasmids in *Har. marismortui* are 33 Kbp, and it is therefore likely that more scaffolding is required.

#### Genome features

The newly-sequenced genomes were similar in size to previously sequenced haloarchaea, ranging from 3.37 to 4.45 Mbp with GC content between 60.3% and 66.3% (Table 2). *Haloferax* genomes were on average 11.6% smaller and encoded 13% fewer protein-coding genes than *Haloarcula* genomes, for an average of 578 fewer genes. The IMG annotation system predicted functions for an average of 66% of protein coding genes in each genome based on matches to a combination of functional annotation databases (COG, Pfam, TIGRfam, InterPro, Gene Ontology, and KEGG) and an IMG native collection of functional roles (IMG terms). Of the predicted protein coding genes in the newly sequenced genomes, 67% matched an entry in the COG database, 51% had one or more significant matches to Pfam domains, and 87% matched an InterPro family or superfamily. The fraction of genes with functional predictions in these genomes is considerably lower than for well-studied bacterial species such as *Escherichia coli* K12 (87%), *Bacillus subtilis* BSn5 (74%), and *Pseudomonas aeruginosa* 2192 (82%) but comparable to previously sequenced haloarchaeal genomes (average 59%).

#### Differential enrichment of COG functional groups in *Haloferax* and *Haloarcula* species

**Figure 2:**
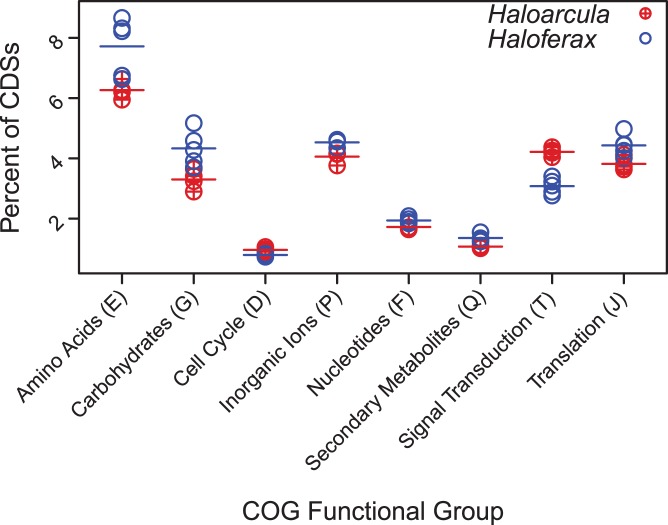
COG enrichment. COGs comprising a significantly different fraction of protein coding genes between *Haloarcula* and *Haloferax* species. Averages for each genus are signified by horizontal bars. Significance was determined with the non-parametric Wilcoxon ranksum test. For p-values and the portion of protein coding genes dedicated to these COGs in each species, see Table S2.

Further investigation of the protein coding complements of *Haloferax* and *Haloarcula* species revealed that several functional categories were differentially enriched between the two genera, as determined by the non-parametric Wilcoxon rank-sum test (see Table S2 for details). These functional categories included groups of genes involved in metabolism and transport of amino acids, carbohydrates, inorganic ions, nucleotides, and secondary metabolites as well as genes involved in cell division, signal transduction and translation (COG categories E, G, P, F, Q, D, T, J respectively). One of the largest differences was in signal transduction, with *Haloferax* species having an average of 68 fewer genes dedicated to this function, amounting to 3.0% of their protein coding potential compared with 4.2% of the protein coding genes in *Haloarcula* species. A large number of signal transduction genes differentially present in *Haloferax* vs. *Haloarcula* genomes were histidine kinases (COG0642, average difference of 22 genes) or CheY-like response regulators (COG2204, average difference of 9 genes). Previous studies have found that, in bacteria, the proportion of an organism’s genes dedicated to certain functions grows disproportionately with increasing genome size [34]. For example, larger bacterial genomes were found to be enriched in signal transduction genes [34]. It is unclear whether these trends extend to the archaeal domain. If so, the relative enrichment of signal transduction genes in *Haloarcula* genomes, in comparison with the smaller genomes of *Haloferax* species, could be explained in terms of a general phenomenon of enrichment for signal transduction in large genomes (Figure 2 and Table S2, see Table 2 for information on genome size). However, species in the clade formed by the *Haloarcula*, *Halomicrobium*, *Halorhabdus*, and *Natronomonas* genera each have approximately 4% of their protein coding genes dedicated to the process of signal transduction, even though the coding potential of these organisms differs by up to 30%, indicating that this may not be a universal principle. In fact, of the nine COG functional groups for which enrichment was previously found to be correlated with genome size [34], only three (nucleotide transport and metabolism (F), signal transduction (T), and translation, ribosome structure and biogenesis (J)) followed the expected trends in *Haloferax* and *Haloarcula* genomes (Table S3), suggesting that trends between functional enrichment and genome size may differ between archaea and bacteria. Alternatively, the trends observed in the *Haloferax* and *Haloarcula* clades may be influenced by historical events in the evolution of these groups and not representative of the Archaea as a whole.

#### Extensive loss of environmental response genes in one Haloferax clade

**Figure 3:**
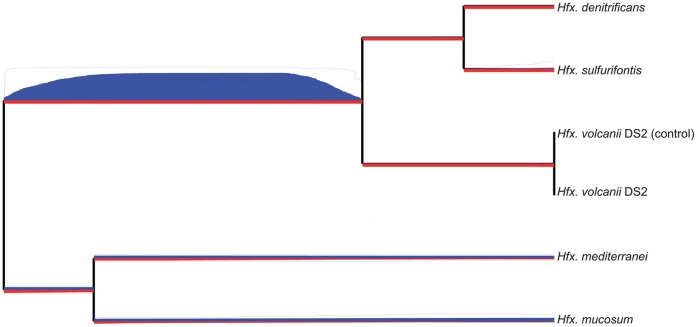
Genomic segment flux in *Haloferax*. GenoPlast was used to infer rates of genomic gain (red) and loss (blue) in the Haloferax lineage, depicted by width of line along phylogeny branch. 95% confidence intervals are represented by bordering thin blue and red lines.

The low number of scaffolds in the final *Haloferax* assemblies allowed inference of historical rates and patterns of genomic segment gain and loss using the GenoPlast software [35] (Figure 3). Although the rate of genome segment gain (red) appears to be consistent throughout the phylogeny, a high rate of loss (blue) is observed in the lineage giving rise to the clade formed by *Hfx. denitrificans*, *Hfx. sulfurifontis* and *Hfx. volcanii*. Genome segment loss in this clade could be a signal of re-organization of the genetic repertoire in response to a major change in environment, habitat, or niche, similar to accelerated gene loss in *Shigella* lineages which have been attributed to shifts in host-interaction lifestyle [35].

**Figure 4:**
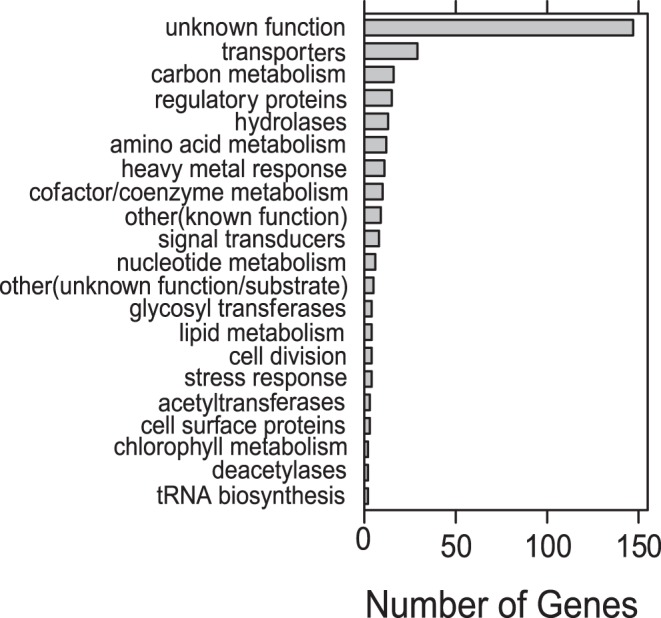
Differential gene gain/loss between two *Haloferax* clades. Number of genes in each category differentially present between the *marismortui-mucosum* and the *denitrificans-sulfurifontis*-*volcanii* clades of *Haloferax*. For details on gene categories see Table S4.

As an independent investigation of gene flux within the *Haloferax* we generated a presence-absence matrix of genes for all sequenced Haloarchaea. A total of 303 protein-coding genes were found to be present in the *Hfx. mucosum*-*Hfx. mediterranei* clade but absent in the other *Haloferax* clade. Of these, about one-half (156 genes) had an annotated function. While ambiguities associated with automated annotation make predicting the precise function of many of these genes uncertain, several broad functional categories were nevertheless highly represented. These included many genes predicted to be involved in transport (32), including of heavy metals (7) as well as a number of predicted transcriptional regulators (14) and signal transducers (8) (Figure 4) (Table S4). Deeper taxon sampling along this lineage will be required in order to more specifically determine the timing of this genomic flux and to decipher whether it has arisen due to loss of selective pressure for environmental dynamic response in the common ancestor of the *Hfx. denitrificans*-*sulfurifontis*-*volcanii* clade, extensive horizontal gene transfer to the common ancestor of the *Hfx. mediterranei-mucosum* clade, or random genetic drift.

### Metabolism

#### Haloarchaeal sugar catabolism by a semi-phosphorylated Entner-Doudoroff pathway lacking a canonical gluconolactonase

As has been discussed previously [13] many Haloarchaea lack an intact Embden-Meyerhof-Parnas pathway for glucose catabolism. In a 2008 review of metabolism in four sequenced haloarchaea, all species were found to be lacking 6-phosphofructokinase (EC 2.7.1.11), which catalyzes the first committed step in glycolysis [13]. The absence of this enzyme was confirmed in the seven novel genomes investigated in this study as well as the fourteen haloarchaeal genomes currently available from NCBI. This suggests that the inability to complete glycolysis via the classical EMP pathway may be a general feature of haloarchaea, although direct metabolite measurements would be required to exclude the possibility that this step may be carried out by an enzyme lacking homology to phosphofructokinase.

A semi-phosphorylated alternative to the Entner-Doudoroff pathway, which bypasses the first six steps in glycolysis, has been previously described for the haloarchaea [8]. The complete enzyme complement for this pathway was found in each of the haloarchaeal genomes investigated with the exception of gluconolactonase (EC 3.1.1.17), which converts D-glucono-1,5-lactone to D-gluconate in the second step of this pathway. This enzyme was absent in all but three of the twenty-one genomes, being present only in the *Haloferax denitrificans-sulfurifontis-volcanii* clade (HVO_B0083 in *Hfx. volcanii*). As previous work has concluded the presence of a functioning semi-phosphorylated ED pathway in *Haloarcula vallismortis* and *Haloferax mediterranei* based upon evidence for activity of key enzymes in this pathway [36], the absence of an annotated gluconolactonase in these genomes was surprising. To determine whether the gene for this enzyme was present and missed during annotation, a BLASTp [37] search was conducted against all haloarchaeal genomes in the NCBI database using as query the predicted gluconolactonase from *Hfx. sulfurifontis*. The six haloarchaeal BLAST hits recovered had relatively high E-values (∼0.001) and low query coverage, with the hit from *Har. marismortui* (rrnAC0850) matching the query with only 64% coverage. The putative gluconolactonase from *Haloarcula marismortui* belongs to the beta-propeller clan (Pfam clan CL0186, E-value = 3×10^−5^), the same clan to which the *Hfx.* gluconolactonases belong. However, this clan contains a large number of families and domains (57 and >200,000 respectively) with a wide variety of functions and it is unclear whether rrnAC0850 and the predicted *Hfx.* gluconolactonases share a function.

To investigate whether these results could stem from a high level of sequence divergence of this enzyme, a BLASTp search against NCBI’s non-redundant protein database was conducted with the same query. The only archaeal match was to *Haloferax volcanii* (HVO_B0083) with many additional high-quality matches to bacterial gluconolactonases (315 bacterial hits with E-value less than or equal to 10^−20^). Although these bacterial matches had low E-values and high query coverage (mean 92%), the percent identity was low (mean 31%). These results suggest that this gene may have been introduced into the *Hfx. denitrificans-sulfurifontis-volcanii* clade via a horizontal transfer event from the Bacteria. The low degree of identity between *Haloferax* and bacterial gluconolactonase may reflect rapid evolution in response to selective pressure for an acidic pI, which is required for protein function in the extremely saline cytoplasm of haloarchaea. Due to the apparent absence of gluconolactonase in other haloarchaea, we propose that haloarchaeal species previously described as using the semi-phosphorylated ED pathway may use an alternative entry of metabolites into this pathway, possibly including a novel mechanism for gluconate production. Further genetic and biochemical work will be required to decipher this unique pathway for sugar metabolism in the haloarchaea as well as the function of the bacterial gluconolactonase-like protein in the *Haloferax denitrificans-sulfurifontis-volcanii* clade.

#### Haloarchaeal species are rich sources of enzymes for biotechnology applications and novel metabolic pathways

Several recent studies have highlighted the unique metabolic capabilities of the haloarchaea and have uncovered enzymes with potential utility in several industrial processes, including biofuels manufacturing. Here we expand upon previous studies by surveying twenty-one haloarchaea for genes of biotechnological importance as well as genes involved in several metabolic processes unique to the haloarchaea.

**Figure 5:**
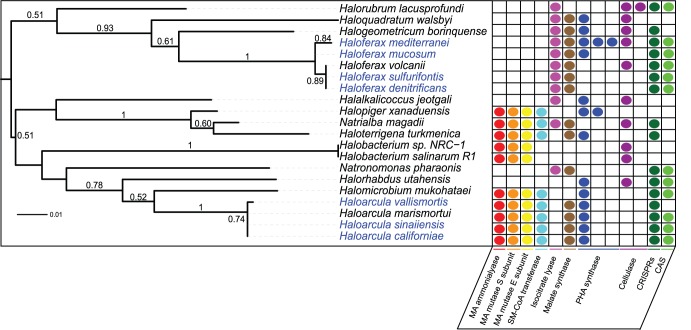
Enzymes of interest in biotechnology and novel metabolism. Gene counts of enzymes discussed in the text superimposed on *rpoB*′′ phylogeny. Methylaspartate ammonia-lyase (EC 4.3.1.2) – MA ammonia-lyase, methylaspartate mutase (EC 5.4.99.1) – MA mutase, succinyl-CoA:mesaconate CoA-transferase – SM-CoA transferase, polyhydroxyalkanoate synthase – PHA synthase, CRISPR associated sequences – CAS. Blue leaf labels indicate genomes sequenced in this study, black leaf labels indicate previously sequenced genomes. CRISPR and CAS data is represented as binary presence/ absence with abundance information provided in Table 3. MA mutase S subunit is also shown as binary although some species have two homologs of this gene, as only one homolog is present at the methylaspartate cycle locus.

Current biofuel production processes depend upon the use of cellulases, which are abundantly distributed in nature. However, the strong ionic liquids increasingly used in biomass pretreatment are inhibitory to many cellulases [11]. The adaptation of haloarchaeal enzymes to high salt concentrations is thought to also confer added structural stability in organic solvents [11], making these organisms ideal candidates for discovery of cellulases useful for biofuels production. This potential was recently confirmed with the discovery of a halotolerant and thermostable cellulase in the haloarchaeon *Halorhabdus utahensis* (*Hu*-CBH1) [11]. Our search of haloarchaeal genomes revealed eleven cellulases distributed broadly across the haloarchaea, with *Haloarcula* species, however, notably lacking these enzymes (Figure 5).

**Table 3:** CRISPR counts.

Organism	CRISPRs	CAS genes
*Haloferax mucosum* ATCC BAA-1512	16	7
*Haloferax denitrificans* ATCC 35960	12	7
*Haloferax sulfurifontis* ATCC BAA-897	10	6
*Haloferax mediterranei* ATCC 33500	5	7
*Haloferax volcanii* DS2	4	6
*Haloarcula californiae* ATCC 33799	6	4
*Haloarcula marismortui* ATCC 43049	5	7
*Haloarcula sinaiiensis* ATCC 33800	3	6
*Haloarcula vallismortis* ATCC 29715	1	0
*Halorubrum lacusprofundi* ATCC 49239	3	11
*Halomicrobium mukohataei* DSM 12286	2	6
*Halorhabdus utahensis* DSM 12940	2	6
*Natronomonas pharaonis* DSM 2160	4	6
*Halobacterium salinarum* R1	0	0
*Halobacterium sp* NRC-1	0	0
*Haloquadratum walsbyi* DSM 16790	0	0
*Halogeometricum borinquense* DSM 11551	1	0
*Halalkalicoccus jeotgali* B3	0	0
*Natrialba magadii* ATCC 43099	2	0
*Haloterrigena turkmenica* DSM 5511	1	0
*Halopiger xanaduensis* SH-6	0	0

Note: Number of CRISPRs is the number of distinct CRISPR clusters.

Haloarchaea have also been explored for their ability to produce polyhydroxyalkanoates (PHA), a potential renewable and biodegradable substitute for petroleum-derived plastics [38]. A recent screen for PHA production in twenty haloarchaeal species detected accumulated PHAs in members of the *Halococcus*, *Halorubrum*, *Natronobacterium*, *Natronococcus*, and *Halobacterium* genera [38]. Our genomic screen showed polyhydroxyalkanoate synthases to be present in *Hfx. mediterranei* and absent in *Halobacterium sp.* NRC-1, *Halobacterium salinarum* R1 and *Hfx. volcanii*, corroborating the results of the previous study. We subsequently experimentally confirmed PHA accumulation in *Halalkalicoccus jeotgali* (Figure S2), demonstrating the power of genomic screens to identify organisms of interest for PHA production. Our genomic screen identified polyhydroxyalkanoate synthase genes in thirteen of the twenty-one species investigated, including all of the *Haloarcula*. These findings suggest that the *Haloarcula* could be a rich source of catalysts for the production of bio-plastics.

The novel metabolic capabilities of haloarchaea include the recently discovered methylaspartate cycle, a unique pathway for assimilation of acetyl-coA derived from metabolism of organic substrates identified in *Har. marismortui* in 2011 [39]. The methylaspartate cycle enables shunting of acetyl-CoA into biosynthesis pathways by bypassing the net decarboxylation steps of the TCA cycle, and as such, functions as an alternative to the glyoxylate cycle. Key enzymes for the methylaspartate cycle, methylaspartate mutase (EC 5.4.99.1) and methylaspartate ammonia-lyase (EC 4.3.1.2), have been proposed to have been gained via horizontal gene transfer from bacteria, where they participate in glutamate fermentation [39]. In *Haloarcula marismortui*, genes involved in the methylaspartate cycle are co-localized on the genome (rrnAC0683-rrnAC0690), with genes rrnAC0684-rrnAC0690 forming an operon, and succinyl-CoA:mesaconate CoA-transferase (rrnAC0683) being upstream of and anti-parallel to the operon [39]. This gene organization is conserved in all *Haloarcula* species, along with *Halomicrobium mukohataei*, *Natrialba magadii*, *Haloterrigena turkmenica*, and *Halopiger xanaduensis*. The gene for succinyl-CoA:mesaconate CoA-transferase was missing in *Halobacterium* species, suggesting that the methylaspartate cycle may not be functional in these organisms. Other haloarchaea were found to possess the key enzymes required for carrying out acetyl-CoA assimilation via the glyoxylate cycle, including *Natronomonas pharaonis*, *Haloquadratum walsbyi*, *Halogeometricum borinquense*, and all *Haloferax* species (Figure 5). However, several haloarchaeal species appear to be missing key enzymes from both the glyoxylate and methylaspartate cycles and it is unclear whether these organisms may utilize an as-yet-unknown pathway for carbon assimilation through the common acetyl-CoA intermediate.

The results of our genomic screens illustrate the power of comparative genomics for discovering patterns in gene distribution and for selecting target organisms or clades in which to conduct searches for genes of functional interest. For instance, we show that the search for polyhydroxyalkanoate synthases would benefit from including the *Haloarcula*, whereas the search for cellulases may be better off omitting this genus.

#### The novel sensory rhodopsin SRIII arose relatively late in haloarchaeal evolution

The opsin family proteins are widespread in the Haloarchaea and serve a number of important roles in the light-dependent physiology of these organisms. Four main classes of haloarchaeal opsins have been previously described: the bacteriorhodopsin H^+^ pump utilizes light energy to establish a proton gradient for ATP production, halorhodopsin serves as a Cl^-^ pump to regulate cytoplasmic osmolarity, and the class 1 and class 2 sensory rhodopsins enable phototactic and photophobic responses to different wavelengths of light. In addition, a third class of sensory opsins with unknown function has been reported for *Haloarcula marismortui* [40] and a variant form of bacteriorhodopsin has been characterized in *Haloarcula marismortui* [41] and in *Haloquadratum walsbyi* [42], [43].

**Figure 6:**
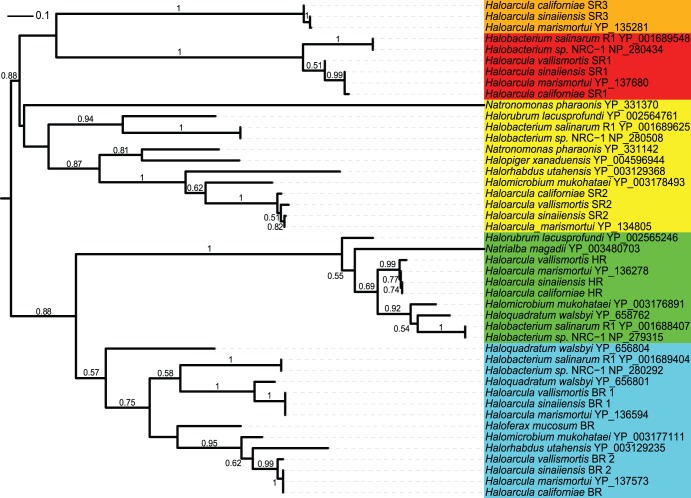
Phylogeny of haloarchaeal opsins. A maximum likelihood tree of the four previously described haloarchaeal opsin families along with the newly described sensory rhodopsin 3, with bootstrap support values above 0.50 shown for 500 bootstrap iterations. Sensory rhodopsin - SR, halorhodopsin - HR, bacteriorhodopsin - BR.

We performed a genomic screen to identify opsin homologs in the twenty-one currently sequenced haloarchaeal genomes. One or more opsins were found in fourteen of the twenty-one genomes, with most species lacking at least one of the four canonical opsin classes. In fact, only *Halobacterium* and *Haloarcula* species were found to possess at least one homolog each of bacteriorhodopsin, halorhodopsin, and sensory rhodopsins 1 and 2. Phylogenetic analysis of opsin sequences obtained from the genomic screen revealed divergent sensory rhodopsins in three species of *Haloarcula* – *Har. californiae*, *Har. sinaiiensis*, and the previously sequenced *Har. marismortui* (rrnAC0559) (Figure 6). The absence of a sensory rhodopsin 3 homolog in *Har. vallismortis* suggests that this gene arose or was introduced into the *Haloarcula* clade after divergence of *Har. vallismortis* from the other three sequenced members of this genus. Alternatively, this gene may be undetected in *Har. vallismortis* due to the relatively high number of contigs in the assembly for this organism. In addition, our screen has revealed homologs of the previously discovered bacteriorhodopsin variant in *Har*. *sinaiiensis* and *Har. vallismortis* in addition to those known in *Haloquadratum walsbyi* (YP_656801) and *Har. marismortui* (YP_136594). The lack of annotation for this gene in *Har. californiae* may be due to the high number of contigs in this assembly. We have also identified, for the first time, an opsin belonging to a member of the *Haloferax* genus, which has previously been found to lack these proteins [44]. Our screen identified a canonical bacteriorhodopsin in the genome of *Haloferax mucosum*, but not in the other *Haloferax* species included in this study.

### DNA Metabolism

#### Several distantly related Haloarchaeal genera possess multiple homologs of eukaryotic proliferating cell nuclear antigen (PCNA)

Proliferating cell nuclear antigen (PCNA), also referred to as DNA sliding clamp in archaea, plays an essential role in many aspects of DNA metabolism, serving as a processivity clamp for the replicative DNA polymerase and also acting as a scaffold for recruitment of proteins with diverse roles in DNA metabolism [45]. Until recently, all Archaea were thought to possess only one PCNA homolog, however, the discovery of two distinct PCNA proteins in *Pyrobaculum aerophilum* (a crenarchaeote) and *Thermococcus kodakaraensis* (a euryarchaeote) has raised the possibility for specialization of redundant copies of this multi-purpose protein [46].

**Figure 7:**
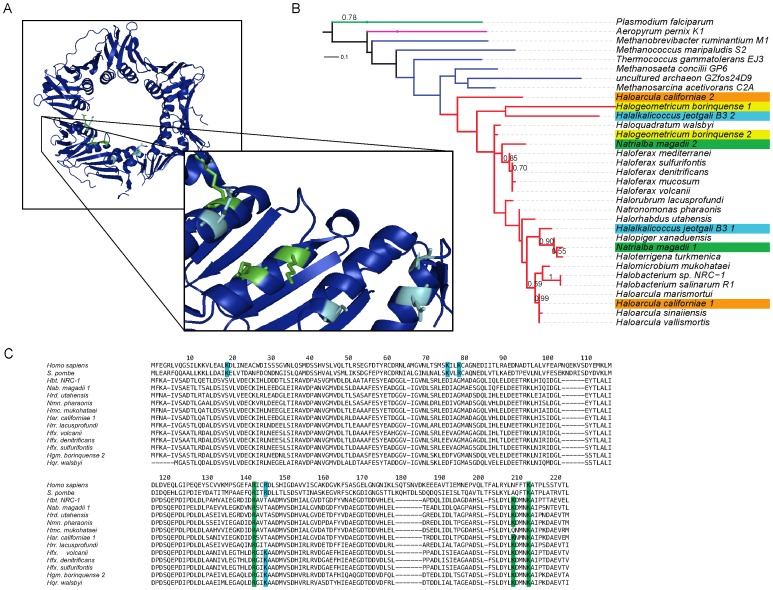
Proliferating cell nuclear antigen (PCNA). (A) Crystal structure of *Haloferax volcanii* PCNA [61] with eukaryotic (light blue) and potential haloarchaeal DNA binding residues (green) shown. (B) Maximum likelihood tree of eukaryotic and archaeal PCNAs with bootstrap support values above 0.50 shown for 500 bootstrap iterations. Branch colors: green – eukarya, purple – crenarchaeota, dark blue – euryarchaeota, red - haloarchaea. Duplicate haloarchaeal PCNAs are distinguished with colored leaves. (C) An alignment of eukaryotic and haloarchaeal PCNA homologs. Residues known to be involved in DNA binding in eukaryotes are shown in light blue, with suspected functionally homologous positions in haloarchaea shown in green.

We have discovered four instances of duplicate PCNAs in the haloarchaea, including the newly sequenced *Har. californiae*. The phylogenetic distribution of these multiple PCNA homologs precludes their origin from a single duplication or horizontal gene transfer event and suggests a more complex history (Figure 7B). By comparing the PCNA phylogeny with that of the molecular marker *rpoB* (see Methods and Materials), it is possible to begin disentangling the evolutionary history of the multiple PCNA homologs. The origin of the second *Natrialba magadii* PCNA (green) has been traced to *Natrialba* phage PhiCh1 (NP_665977) indicating that it has arisen from HGT. The origins of the additional PCNA homologs of *Har. californiae* (orange), *Halogeometricum borinquense* (yellow, Hbor_39470), and *Halalkalicoccus jeotgali* (green, HacjB3_05215) are less clear. These sequences may have arisen through HGT from within the haloarchaea or from a closely related euryarchaeal clade. Additionally, the branching position of the second *Halogeometricum borinquense* PCNA homolog could be consistent with origin in a duplication and divergence event (Figure 7). Experimental work will be required in order to determine whether the multiple PCNA homologs within these species are functional, and if so, whether specialization has occurred to partition the many functions of PCNA among these homologs.

Previous work has shown *Hfx. volcanii* PCNA to lack three of four amino acid residues crucial for interaction with DNA in eukaryotic PCNA [46], [47]. These basic residues line the central pore of PCNA and are thought to interact with the phosphate backbone of DNA, allowing sequence independent binding [46]. Investigation of PCNAs from the twenty-one currently sequenced haloarchaea reveals that even the one basic residue preserved in *Haloferax volcanii* (Lys 143) is not conserved among haloarchaea, with about half of haloarchaeal PCNAs having a Lys Thr mutation at this position. We also note, however, that the majority of haloarchaeal PCNA sequences share a different set of three basic amino acid residues, also located in the central pore in analogous positions to three of the eukaryotic residues known to be involved in DNA binding (two on sequential turns of an alpha helix, with the third on an adjacent alpha helix). We hypothesize that these residues may interact with DNA in a manner similar to that observed in eukaryotes. Subsequent genetic and structural analysis will be required in order to confirm the importance of these residues to DNA binding.

#### CRISPRs and CRISPR-associated (Cas) genes are not universally conserved in the Haloarchaea

The Clustered Regularly Interspaced Short Palindromic Repeat (CRISPR) system is a recently discovered method of viral immunity present in a variety of bacterial and archaeal genomes. Although previous studies have reported CRISPR arrays in approximately 40% of tested bacterial and 90% of archaeal genomes [48]–[50], the relative scarcity of sequenced archaeal genomes has limited the number of Archaea included in these studies. As such, the distribution and importance of CRISPR-mediated immunity systems in Archaea remains undetermined.

The twenty-one sequenced haloarchaeal genomes available at the time of this study were searched for CRISPRs and CRISPR-associated (Cas) genes. Twelve of the twenty-one species were found to possess both CRISPRs and one or more Cas genes, including all *Haloferax* and all but one *Haloarcula* species (Table 3 and Figure 5). However, five of the tested haloarchaea (two closely related strains of *Halobacterium* and one species from each of the genera *Haloquadratum*, *Halopiger*, and *Halalkalicoccus*) had no predicted CRISPRs or Cas genes, supporting previous work [49], [50] which had noted the absence of CRISPRs in some haloarchaeal species, and calling into question the necessity of the CRISPR system for phage resistance in this clade. Intriguingly, four species - *Haloterrigena turkmenica*, *Haloarcula vallismortis*, *Halogeometricum borinquense*, and *Natrialba magadii* - were found to be lacking any of the known CRISPR-associate genes, even though they were predicted to possess one or more CRISPR loci. As the function of many of the Cas genes and their contributions to the CRISPR/Cas phage-defense system are still unknown [51], it is possible that more genes involved in this process remain to be discovered. Alternatively, these CRISPRs may be either vestigial, with the Cas genes having been lost from the genome, or false discoveries by the CRISPR finding software. Recent work has shown the existence of a vestigial CRISPR locus in *Haloquadratum walsbyi* DSM 16790, which was not identified in our analysis [52]. The closely related strain, *Haloquadratum walsbyi* C23 was found to possess three CRISPR loci, including one homologous to the vestigial CRISPR locus in strain DSM 16790 [52]. The presence of vestigial CRISPR loci in these genomes could support the notion that the CRISPR system may not be an essential survival mechanism in the haloarchaea, but could also reflect the sheltered life of laboratory strains, which lack selective pressure to retain resistance to viral infection. This second explanation is supported by the loss of a functioning CRISPR system in *Haloquadratum walsbyi* DSM 16790, which was isolated via two years of serial enrichment, as compared with strain C23 which was isolated over a three-week period via the extinction dilution method [52].

The direct repeats (DRs) of the CRISPR array have been previously shown to be highly conserved across CRISPR loci both within and between closely related species of haloarchaea [48], [53]. We found that fourteen of the haloarchaeal species with predicted CRISPRs possessed one or more with highly similar DRs, including two of the four species with no predicted Cas genes (*Halogeometricum borinquense* and *Natrialba magadii*) but excluding *Haloterrigena turkmenica* and *Har. vallismortis*. This suggests that *Halogeometricum borinquense* and *Natrialba magadii* may have gained CRISPR loci in isolation from Cas genes through HGT with other haloarchaea, or lost Cas genes due to disuse of the CRISPR/Cas system as discussed above. In contrast, it appears that *Haloterrigena turkmenica* and *Har. vallismortis* may have gained their CRISPR loci through more distant HGT events.

**Figure 8:**
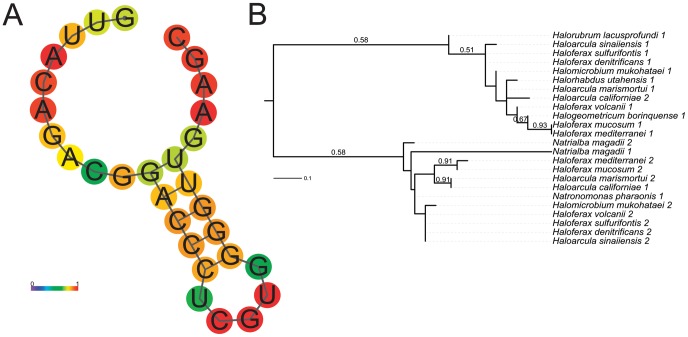
Clustered Regularly Interspaced Short Palindromic Repeats (CRISPRs). (A) Secondary structure of a sub-group of highlyconserved CRISPR direct repeats (DRs) predicted with RNAfold with percent conservation shown as heatmap. (B) Maximum likelihood tree of DRs with bootstrap support values above 0.50 shown for 500 bootstrap iterations.

Alignment of the conserved haloarchaeal DRs shows many highly conserved positions and a few positions (mainly in the center of the repeat) with no apparent conservation. This conservation pattern suggests a conserved stem-loop in the secondary structure, which was confirmed with the program RNAFold [54]–[56] (Figure 8). Predicted stem-loop structures for DRs have been reported previously and are thought to be important for CRISPR function [48].

#### Independent expansions of transcription factor families suggests genera-specific adaptations to environmental fluctuations encountered in niches occupied by each genus

Archaeal transcription initiation is dependent upon two general transcription factors (GTFs) orthologous to eukaryotic TATA-binding protein (TBP) and transcription factor II B (TFIIB, known as TFB in the Archaea) [57]. The presence of multiple copies of one or both of these GTFs in many species of archaea, and especially in haloarchaea, has led to speculation that archaea may utilize different combinations of TBPs and TFBs in a promoter-specific manner to differentially regulate gene expression in a manner analogous to the function of sigma factors in bacteria. Work in the model haloarchaeon *Halobacterium sp*. NRC-1, which with six TBP and seven TFB homologs is the most GTF-rich archaeal species known, has shown promoter-specific binding of individual GTFs leading to differential regulation of transcription in different simulated environments [58], [59].

**Figure 9:**
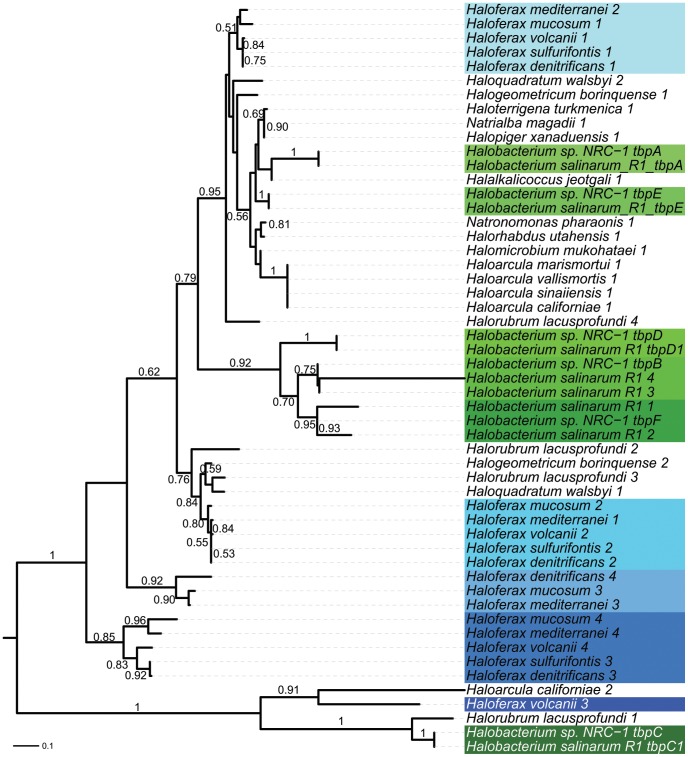
Independent expansion of the TATA-binding protein family in two haloarchaeal genera. A maximum likelihood tree of TATA-binding protein (TBP) homologs identified by RAST with bootstrap support values above 0.50 shown for 500 bootstrap iterations. Successive duplications are shown in darkening shades of green (*Halobacterium*) or blue (*Haloferax*.)

Our analysis reveals that, although TBP and TFB expansions are widespread across the haloarchaea (Table 4), several of these expansions appear to have taken place independently in individual haloarchaeal lineages, rather than being vertically inherited from the common ancestor of haloarchaea. Specifically, we have uncovered an expansion of the TBP family in the *Haloferax* which is eclipsed in number of TBP paralogs only by *Halobacterium*, with most *Hfx.* species possessing four distinct TBP homologs. Phylogenetic analysis reveals that the dramatic expansion of the TBP family in these two genera are independent, with the *Haloferax* and *Halobacterium* TBPs being orthologous, not paralogous, with one another (Figure 9). Additionally, whereas the *Haloferax* TBPs appear to have arisen by a series of duplication events from a single ancestral copy, the clustering pattern of the *Halobacterium* TBPs suggests a series of sequential duplications (especially in the *tbpD*, *tbpB*, and *tbpF* cluster). Hence, although the presence of multiple TBPs in each genus indicates a sophisticated method for differential regulation of transcription initiation, we expect these mechanisms to be quite different from one another, involving different binding motifs and different sets of co-regulated genes in the two genera. In line with the predicted role for GTFs in niche adaptation predicted by Turkarslan et al [59], these expansions have likely arisen to enable differential regulation in response to the specific environmental challenges faced by these organisms in their respective environments.

**Table 4:** General transcription factor (GTF) counts.

Organism	TBPs	TFBs
*Haloferax mucosum*	4	7
*Haloferax denitrificans*	4	8
*Haloferax sulfurifontis*	3	9
*Haloferax mediterranei*	4	8
*Haloferax volcanii*	4	9
*Haloarcula californiae*	2	8
*Haloarcula marismortui*	1	8
*Haloarcula sinaiiensis*	1	8
*Haloarcula vallismortis*	1	7
*Halorubrum lacusprofundi ATCC 49239*	4	9
*Halomicrobium mukohataei DSM 12286*	1	4
*Halorhabdus utahensis DSM 12940*	1	7
*Natronomonas pharaonis DSM 2160*	1	7
*Halobacterium salinarum R1*	8	9
*Halobacterium sp NRC-1*	6	7
*Haloquadratum walsbyi*	2	8
*Halogeometricum borinquense*	2	8
*Halalkalicoccus jeotgali*	1	7
*Natrialba magadii*	1	7
*Haloterrigena turkmenica*	1	6
*Halopiger xanaduensis*	1	9

### Conclusions

Comparisons of the twenty-one currently available haloarchaeal genomes have revealed dynamics of genome evolution at scales ranging from horizontal transfer and duplication of individual genes to major gene loss events and large-scale expansion of functional groups. Deep sequencing of the *Haloarcula* and *Haloferax* genera has further enriched our ability to conduct genomic analyses of the haloarchaea, for example, by enabling identification of genomic flux events at a finer time-scale than previously possible. In addition to providing a broad outline of the history of genomic evolution in this clade, these comparisons have identified several areas of specific interest for future investigation into the unique metabolic and regulatory capabilities of the haloarchaea.

## Methods

### Strain Growth and DNA Isolation

Strains were acquired as desiccated cells from the American Type Culture Collection (ATCC) in Manassas, Virginia and were rehydrated in recommended media according to ATCC protocols. Strains were grown to stationary phase at 37°C in liquid culture and genomic DNA harvested with the Wizard Genomic DNA purification (Promega).

### Sequencing and Assembly

Fragment libraries were constructed for eight species of the family Halobacteriacea, three from the genus *Haloarcula* (*Har. californiae*, *Har. sinaiiensis*, *Har. vallismortis*) and five from the genus *Haloferax* (*Hfx. denitrificans*, *Hfx. mediterranei*, *Hfx. mucosum*, *Hfx. sulfurifontis*, and *Hfx. volcanii*), and sequenced on a single GS FLX Titanium run following standard protocols (454 Life Sciences - http://454.com/). *Hfx. volcanii* was included as a sequencing control, as its genome had been completed previously [19]. Additionally, for *Har. sinaiiensis* and *Hfx. mediterranei*, 8 Kb pair-end libraries were constructed and the terminal 100 bp of each end was sequenced, according to standard protocols. The paired-end information and any trimming information are specified using annotation strings on the description line of the reads. Reads were assembled using the Genome Sequencer De Novo assembler (454 Life Sciences - http://www.my454.com/). Final assemblies are available at our website [60] and as Dataset S3.

### Annotation and Metabolic Modeling

Genomes were annotated with Rapid Annotation Using Subsystems Technology (RAST) [61] as well as with the Integrated Microbial Genomes (IMG) system [62], each of which proved useful for particular types of analyses. The presence and absence of specific genes was investigated by searching RAST output GenBank files using in-house scripts available at our website [60] and in the Supplementary Online Material. Where appropriate, annotation accuracy was confirmed with BLASTp searches [37] and searches of the Pfam database [63]. RAST-annotated genomes can be accessed through the PubSEED identifiers listed in Table 1. Overall genome features and distribution of COG functional groups was determined from IMG annotations. These annotations have been made publicly available, and can be accessed through the Integrated Microbial Genomes – Genome Encyclopedia of Bacteria and Archaea (IMG/GEBA) system at http://img.jgi.doe.gov/cgi-bin/geba/.

### Syntenic Halophilic Tribes (SHTs) Matrix

In order to determine phylogenetic distribution of haloarchaeal genes, a gene presence/absence matrix was constructed by the following process. Independent multi-genome alignments were made for the *Haloferax* and *Haloarcula* genera using the whole genome alignment method progressiveMauve [64]. The contigs for each alignment were reordered to match the published genomes of *Haloferax volcanii* [19] and *Haloarcula marismortui* [18], respectively, using Mauve’s built-in contig reordering program (Figures S3 and S4). Sets of functionally homologous genes (orthologs), referred to hereafter as Syntenic Halophile Tribes (SHTs), were determined from alignments and joined by the following process. The proteins in each SHT from the *Haloferax* alignment were searched against all proteins in each SHT from the *Haloarcula* genomes using BLAST [37] and a bit score for each pair of SHTs was calculated by averaging the bit scores from each BLAST hit. A traditional reciprocal best hit (RBH) BLAST approach was used to produce one-to-one mappings between SHTs in the two genera. Each joined SHT was assigned a function using the most commonly occurring functional annotation of the protein products of the genes in the SHT. This resulted in a set of 398 SHTs present in all nine genomes.

Hidden Markov Models (HMMs) were generated for each SHT using HMMER 3, resulting in 13,276 HMMs. The 1,303 completed archaeal and bacterial genomes available from NCBI as of March 15, 2011 were downloaded and a single genome from each genus selected at random, resulting in 396 genomes. Each SHT HMM was searched against these 396 genomes and the eight halophile genomes generated for this study using HMMER 3. Each gene was counted as belonging to the HMM if it had an E-value below 0.0001 and the hit covered greater than 80% of the length of both the gene and the HMM. If a gene hit more than one HMM it was counted only for the HMM with the best E-value. These hits were then used to generate a 13,276 x 405 presence/absence matrix. The genomes and HMMs were clustered using the ‘ctc’ library in R [65] with manhattan distance and complete linkage clustering. The clustering was viewed with the Java Treeview program [66]. Cluster file can be accessed at our website [60] and as Dataset S1 and Figure S5.

### Phylogeny Reconstruction

Three methods were used to determine the phylogenetic relationships among the newly sequenced haloarchaea. First, a tree was constructed using a set of twenty-eight highly conserved marker genes identified with Amphora [67]. HMMs were constructed for these marker genes based on sequence data from sixty-two previously sequenced Archaea. HMMER 3.0 [68] was used to identify the marker genes within the fourteen previously sequenced and seven newly sequenced haloarchaeal genomes. For each of the twenty-eight marker genes, a multiple alignment of the twenty-one haloarchaeal gene sequences was constructed using MUSCLE [69]. The alignments were concatenated into a super-alignment and a phylogenetic tree was constructed through the online phylogenetic workflow Phylogeny.fr [70] using PhyML with 500 bootstrap iterations and visualized with iTOL [71], [72] (Figure S6).

A second method was also used wherein the proteins within each of the 398 SHTs conserved across the seven newly-sequenced haloarchaeal genomes and the two previously sequenced representatives of the *Haloarcula* and *Haloferax* clades were aligned using MUSCLE [69]. Individual phylogenetic trees were inferred for each multiple sequence alignment using Mr. Bayes [73] and a primary concordance tree was constructed with BUCKy [74] (Figure S7). Each clade in the concordance tree is labeled with a concordance factor that represents the fraction of support for the clade across the 398 individual gene trees.

Thirdly, a tree was constructed based on the commonly used molecular marker *rpoB* (in this case subunit *rpoB’’*) [75] using the Phylogeny.fr workflow [70] with the multiple alignment program MUSCLE run in full mode and PhyML with 500 bootstrap iterations (Figure 5). The branching order of the *Haloarcula* and *Haloferax* species were identical in these three trees, with the exception that polytomies were formed by the *Har. californiae*-*sinaiiensis*-*marismortui* and the *Hfx. volcanii*-*sulfurifontis*-*denitrificans* clades in the *rpoB’’* tree. As only 36.8% of conserved genes supported the branching order shown for the *Har. californiae*-*sinaiiensis*-*marismortui* clade in the BUCKy and Amphora trees, it is not surprising that the *rpoB’’* tree failed to resolve the branching order of these species. Presence/absence of particular genes of interest was determined by searching RAST-annotated GenBank files using in-house scripts, available at our website [60] and superimposed onto the *rpoB’’* tree.

### Gene Gain/loss

The multiple genome alignment of six *Haloferax* genomes (including a re-sequenced *Hfx. volcanii* DS2) was used to infer historical rates and patterns of segmental gain and loss using the GenoPlast software [35]. GenoPlast uses a Bayesian compound Poisson process model to jointly estimate rates of segmental gain and loss, along with the individual gain and loss events along a phylogenetic tree. GenoPlast requires a fixed tree topology, which we estimated using the Unweighted Pair Group Method with Arithmetic Mean (UPGMA) in Phylip [76] on the basis of single nucleotide differences present in the core genome multiple alignment.

### Opsins

Opsin homologs were gathered from the RAST-annotated GenBank files using in-house scripts available at our website [60]. Highly divergent sequences were discarded after confirming a lack of Pfam matches to Bacteriorhodopsin-like protein domains (Clan CL0192). A phylogenetic tree was constructed using the Phylogeny.fr workflow [70] with the multiple alignment program MUSCLE run in full mode, and PhyML with 500 bootstrap iterations. The resulting tree was visualized in iTOL [71], [72].

### PCNAs

PCNA homologs were gathered from the RAST-annotated GenBank files using in-house scripts, available at our website [60] and searched against the Pfam database [63] to determine domain architecture. Predicted homologs with significant matches (E-value <0.001) to both PCNA_N and PCNA_C were retained for further analysis. Homologs with insignificant matches to one or both domains were also retained, provided both domains were found, due to the low combined likelihood of finding both domains by chance. For example, *Halalkalicoccus jeotgali* possesses a predicted homolog with insignificant matches to both PCNA_N (E-value  = 0.0011) and PCNA_C (E-value  = 0.17). The combined probability of finding both of these domains by chance is 0.000187, which is below the significance threshold. An alignment was constructed and phylogeny determined for PCNA homologs from the twenty-one sequenced haloarchaea, along with other euryarchaeota, crenarchaeota and eukaryotes using the Phylogeny.fr workflow [70] with the multiple alignment program MUSCLE run in full mode, standard alignment curation with Gblocks, and PhyML with 500 bootstrap iterations. The resulting phylogenetic tree was visualized in iTOL [71], [72]. For full alignment of PCNA homologs see Dataset S2. The pdb file for *Haloferax volcanii* PCNA structure (PDB ID 3IFV) [47] was downloaded from the RCSB Protein Data Bank [77], and manipulated in MacPyMOL [78].

### CRISPRs

CRISPRs were identified for the twenty-one haloarchaeal genomes included in this study using the PILER-CR CRISPR prediction program [79]. The number of CRISPR associated (Cas) genes per genome was determined by searching RAST output GenBank files using in-house scripts available at our website [60]. CRISPRs with closely related DRs were manually curated from a multiple sequence alignment generated with rCoffee [80] and a phylogenetic tree was generated from these DRs using the Phylogeny.fr workflow [70] with the multiple alignment program MUSCLE run in full mode and PhyML with 500 bootstrap iterations. The resulting tree was visualized in iTOL [71], [72]. RNA secondary structure of DRs was determined using the program RNAfold [54]–[56].

### General Transcription Factors

Protein sequences of the predicted general transcription factors (GTFs) TATA-binding protein (TBP) and transcription factor B (TFB) were extracted from the RAST-annotated GenBank files using in-house scripts, available at our website [60] and manually inspected in the multi-alignment viewer Jalview [81], [82]. Highly divergent sequences and sequences representing identical duplicate GTFs were excluded from further analysis. A phylogenetic tree was then constructed for the TBPs using the online phylogenetic analysis pipeline Phylogeny.fr [70] with the alignment program MUSCLE and PhyML with 500 bootstrap iterations. The resulting phylogenetic tree was visualized in iTOL [71], [72].

### Polyhydroxyalkanoate (PHA) Staining

Acid-hydrolyzed rice hull obtained from MicroMidas (Sacramento) was neutralized to pH ∼7 and inoculated with *Halalkalicoccus jeotgali*. Cells were grown to confluence, and washed twice with PBS. Several µL of cell suspension was smeared on a glass slide, air dried, heat fixed, stained with Nile Blue A for 10 min at 55 ^o^C, washed with water and glacial acetic acid, stained with DAPI Gold Bond (R), covered with glass coverslip, dried overnight, and sealed with nail polish. Slides were visualized on a Leica DM600B compound microscope with a 100×objective. PHA and DNA were visualized using the Y3 and A4 filtercubes, respectively.

## Supporting Information

Figure S1 **Scaffold completeness in** ***Haloferax mediterranei*** **.** Results of PCR-based test for circularization of scaffolds. Scaffold 2 (A), scaffold 4 (B), scaffold 5 (C), scaffold 3 (D), scaffold 1 (E), 2-log ladder (L). Approximate positions of PCR products from scaffolds B, C and D are marked along the Y axis. See Table S1 for primer sequences.
(EPS)

Figure S2 ***Halalkalicoccus jeotgali*** **PHA stain.** Polyhydroxyalkanoate (PHA) granules (red) and DNA (blue) in heat-fixed *Halalkalicoccus jeotgali* visualized with Nile Blue A and DAPI, respectively, under 100×objective.
(EPS)

Figure S3 **Multi-genome alignment of** ***Haloferax*** **genomes.** Whole genome alignment of *Haloferax* genomes with contigs re-ordered to reflect order of published *Haloferax volcanii* genome. Colored blocks are regions of predicted homology. Blocks lying below the center line in each panel are in reverse orientation with respect to *Hfx. volcanii* genome. Height of vertical bars within each colored block denote conservation within each homologous region.
(PDF)

Figure S4 **Multi-genome alignment of** ***Haloarcula*** **genomes.** Whole genome alignment of *Haloarcula* genomes with contigs re-ordered to reflect order of published *Haloarcula marismortui* genome. Colored blocks are regions of predicted homology. Blocks lying below the center line in each panel are in reverse orientation with respect to *Hfx. marismortui* genome. Height of vertical bars within each colored block denote conservation within each homologous region.
(PDF)

Figure S5 **Presence/absence matrix of Syntenic Halophile Tribes (SHTs).** Presence (red) and absence (black) of 13,276 hidden Markov models in 405 genomes including the eight genomes generated for this study as well as one genome randomly selected from each of the 396 genera in the NCBI genomes database. Matrix available as.cdt file through our website and as Dataset S1.
(PNG)

Figure S6 **Amphora tree.** Maximum likelihood tree based on twenty-eight highly conserved molecular marker genes identified by Amphora. Bootstrap support values over 0.50 shown for 500 bootstrap iterations.
(EPS)

Figure S7 **BUCKy tree.** Concordance tree constructed with Mr. Bayes from individual gene trees for all 398 SHTs conserved across *Haloferax* and *Haloarcula* species. Branch support values represent the percentage of individual gene trees for which each clade is observed.
(PDF)

Table S1 **Primers for scaffold circularization experiment.** Primer sequences for PCR-based test of scaffold completeness in *Hfx. mediterranei*.
(XLSX)

Table S2 **COG enrichment.** Percent CDSs assigned to COG groups in each species. Genera-wide means and P-values from Wilcoxon rank-sum test are shown.
(XLSX)

Table S3 **Comparison of COG functional group enrichment with study of Konstantinidis et al.** [**34**] **.** P-values shown are from Wilcoxon rank-sum test.
(XLSX)

Table S4 **List of genes differentially present in two** ***Haloferax*** **clades.** Annotations associated with genes present in the *Hfx. mucosum*-*Hfx. mediterranei* clade but absent in the *Hfx. volcanii-denitrificans-sulfurifontis* clade according to the Syntenic Halophilic Tribes presence/absence matrix (Dataset S1).
(XLSX)

Dataset S1 **Syntenic Halophilic Tribes matrix.**
(CDT)

Dataset S2 **Full alignment of Proliferating Cell Nuclear Antigen (PCNA) homologs.** Untrimmed alignment of sixty-one PCNA homologs from fifty-seven archaeal and eukaryotic species constructed with MUSCLE.
(TXT)
