# Supplementary figures and images for: Sequencing of Seven Haloarchaeal Genomes Reveals Patterns of Genomic Flux
Source: PLoS One. 2012 Jul 24;7(7):e41389. doi: 10.1371/journal.pone.0041389 (PMC3404096; doi:10.1371/journal.pone.0041389)

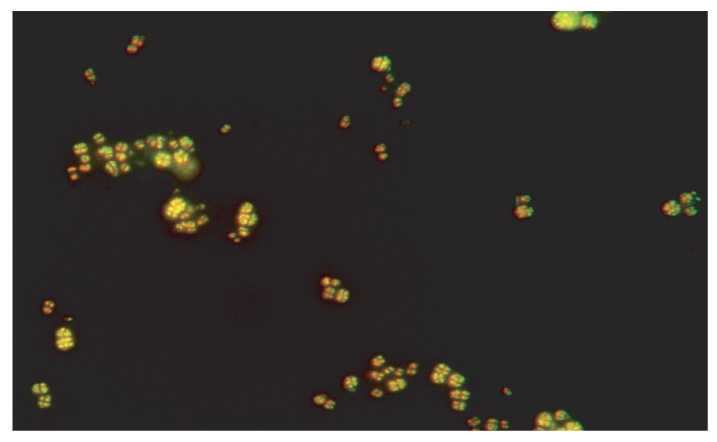

Supplement: Figure S2 — Halalkalicoccus jeotgali PHA stain. Polyhydroxyalkanoate (PHA) granules (red) and DNA (blue) in heat-fixed Halalkalicoccus jeotgali visualized with Nile Blue A and DAPI, respectively, under 100×objective. (EPS) [file pone.0041389.s002.tif]

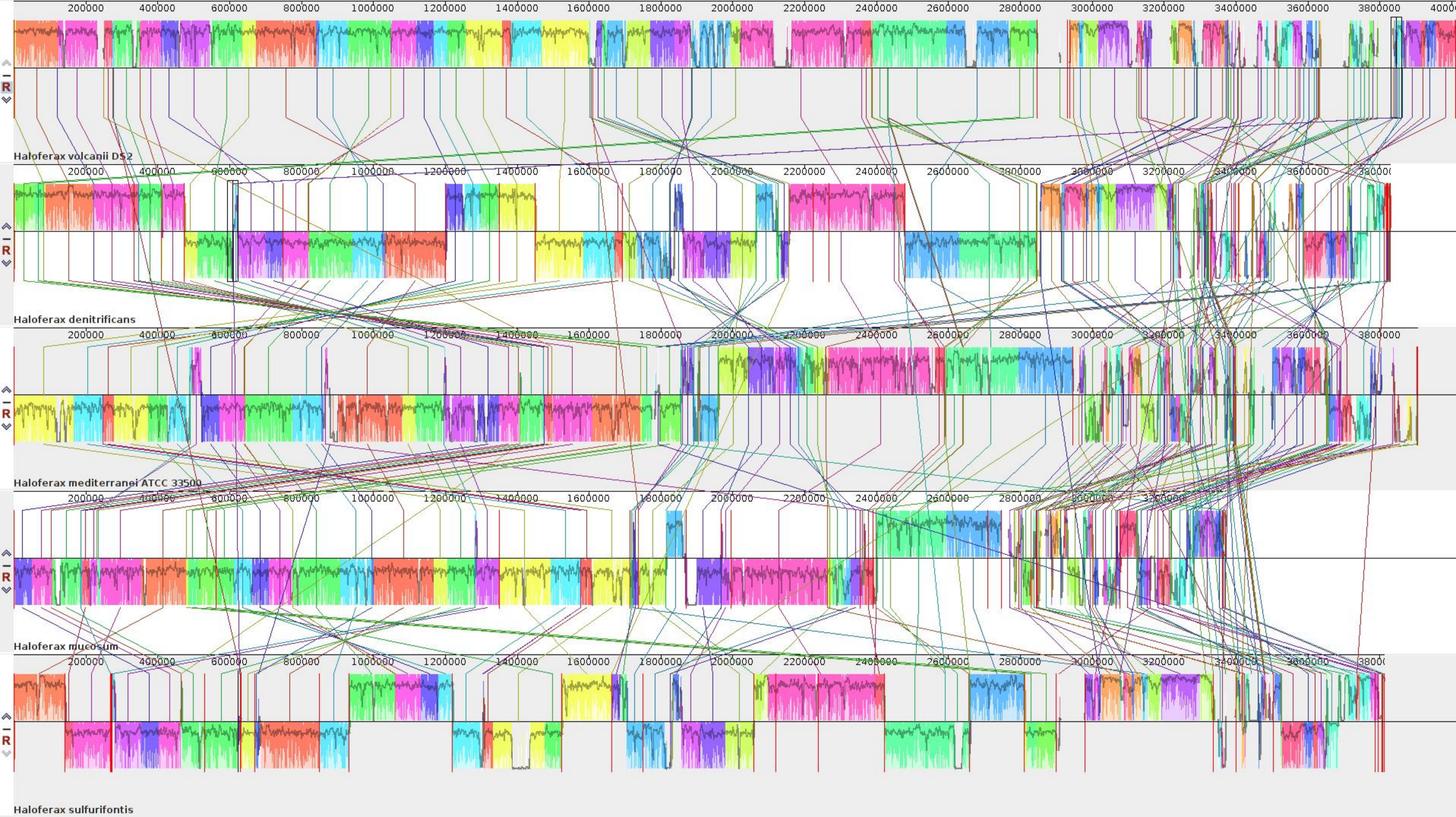

Supplement: Figure S3 — Multi-genome alignment of Haloferax genomes. Whole genome alignment of Haloferax genomes with contigs re-ordered to reflect order of published Haloferax volcanii genome. Colored blocks are regions of predicted homology. Blocks lying below the center line in each panel are in reverse orientation with respect to Hfx. volcanii genome. Height of vertical bars within each colored block denote conservation within each homologous region. (PDF) [file pone.0041389.s003.pdf]

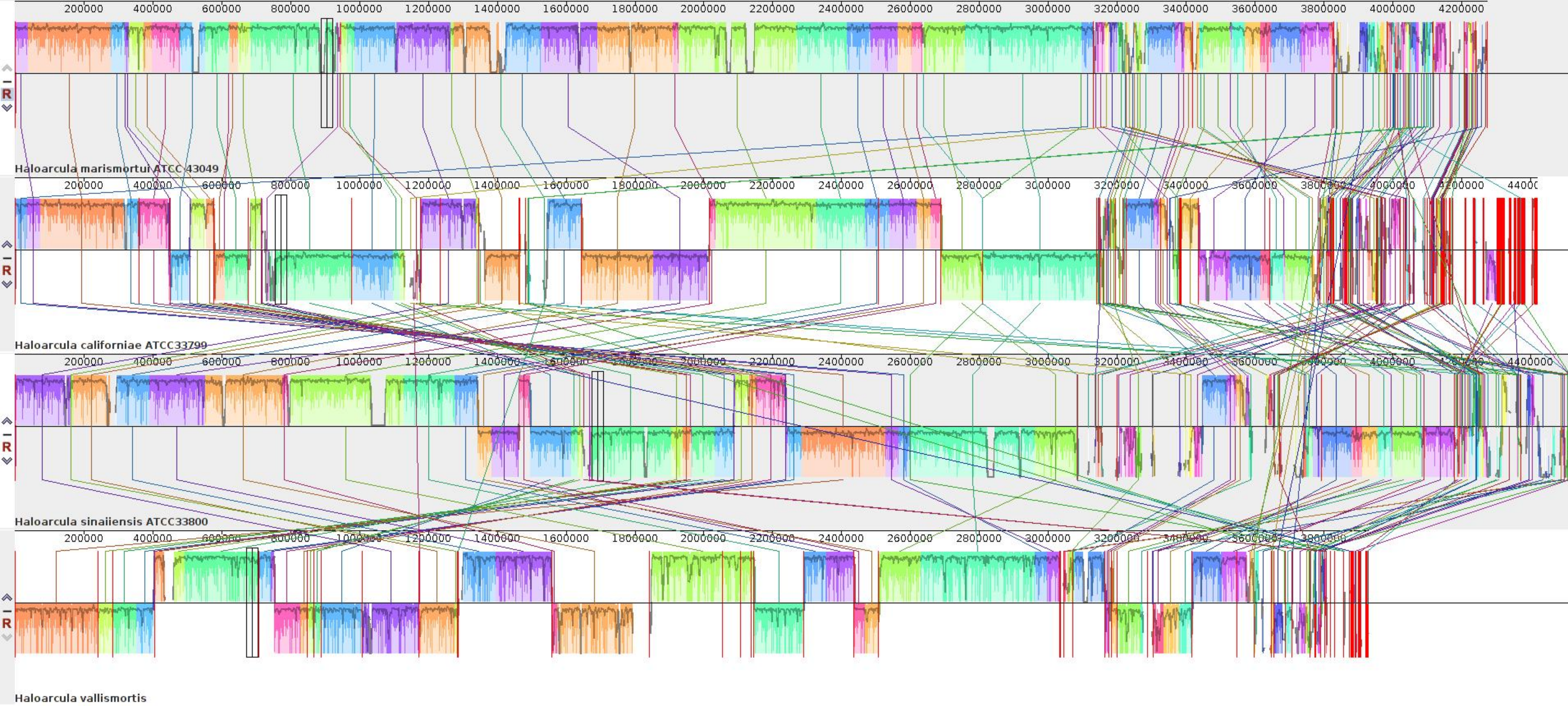

Supplement: Figure S4 — Multi-genome alignment of Haloarcula genomes. Whole genome alignment of Haloarcula genomes with contigs re-ordered to reflect order of published Haloarcula marismortui genome. Colored blocks are regions of predicted homology. Blocks lying below the center line in each panel are in reverse orientation with respect to Hfx. marismortui genome. Height of vertical bars within each colored block denote conservation within each homologous region. (PDF) [file pone.0041389.s004.pdf]

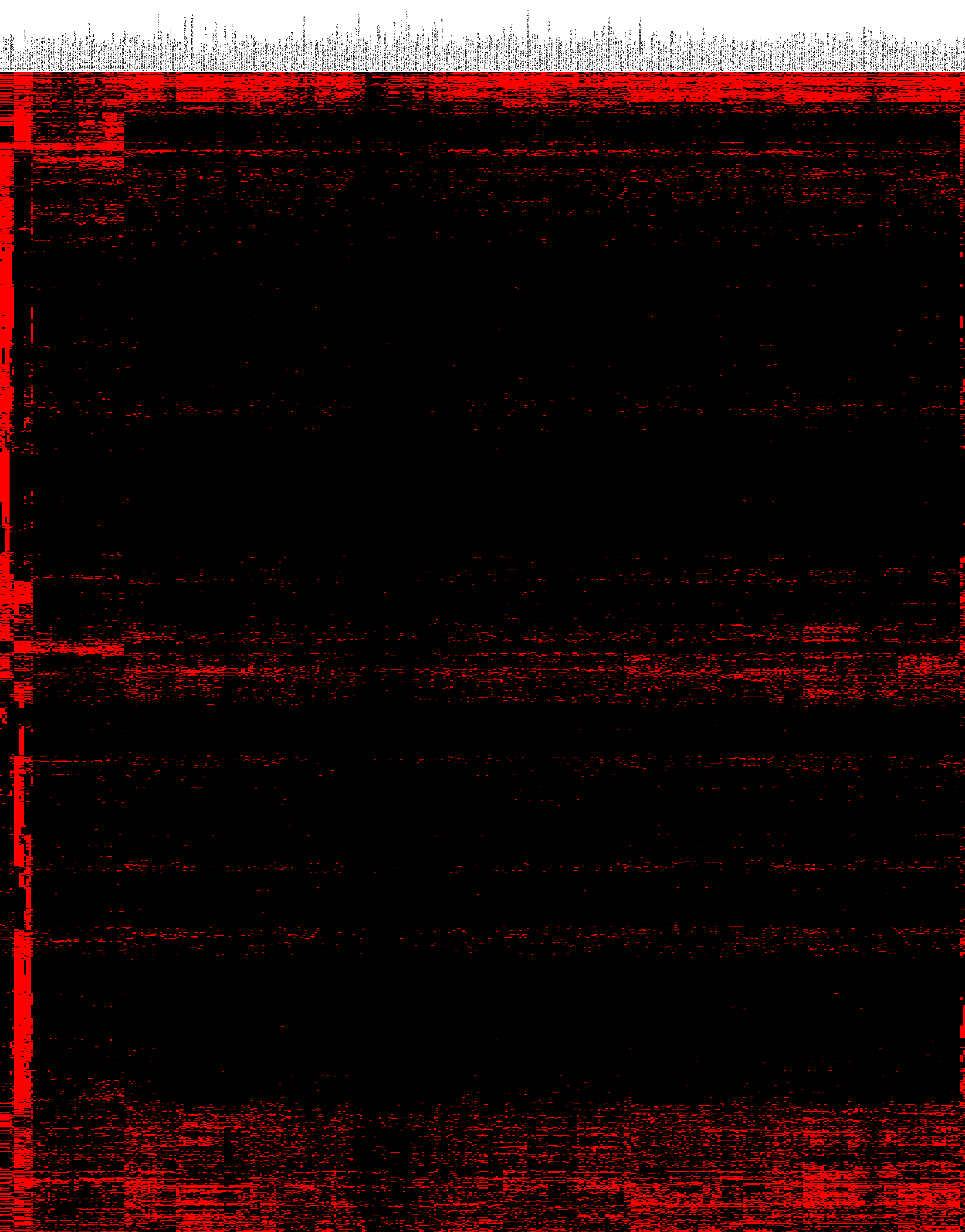

Supplement: Figure S5 — Presence/absence matrix of Syntenic Halophile Tribes (SHTs). Presence (red) and absence (black) of 13,276 hidden Markov models in 405 genomes including the eight genomes generated for this study as well as one genome randomly selected from each of the 396 genera in the NCBI genomes database. Matrix available as.cdt file through our website and as Dataset S1. (PNG) [file pone.0041389.s005.png]

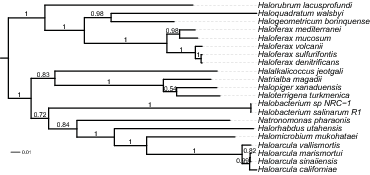

Supplement: Figure S6 — Amphora tree. Maximum likelihood tree based on twenty-eight highly conserved molecular marker genes identified by Amphora. Bootstrap support values over 0.50 shown for 500 bootstrap iterations. (EPS) [file pone.0041389.s006.tif]

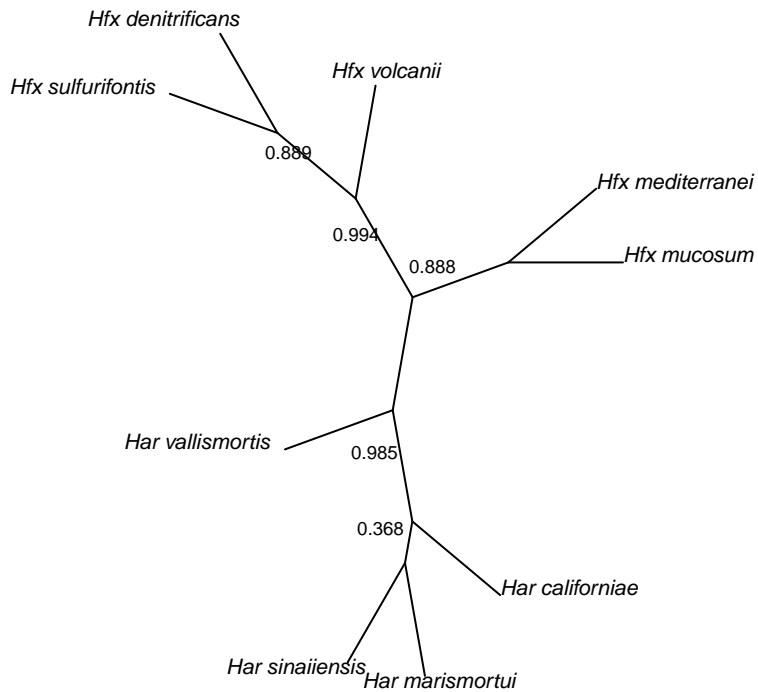

Supplement: Figure S7 — BUCKy tree. Concordance tree constructed with Mr. Bayes from individual gene trees for all 398 SHTs conserved across Haloferax and Haloarcula species. Branch support values represent the percentage of individual gene trees for which each clade is observed. (PDF) [file pone.0041389.s007.pdf]
